# Coronary Plaque Burden, as Determined by Cardiac Computed Tomography, in Patients with Myocardial Infarction and Angiographically Normal Coronary Arteries Compared to Healthy Volunteers: A Prospective Multicenter Observational Study

**DOI:** 10.1371/journal.pone.0099783

**Published:** 2014-06-17

**Authors:** Elin B. Brolin, Tomas Jernberg, Torkel B. Brismar, Maria Daniel, Loghman Henareh, Jonaz Ripsweden, Per Tornvall, Kerstin Cederlund

**Affiliations:** 1 Department of Radiology, Karolinska University Hospital Huddinge and Department of Clinical Science, Intervention and Technology, Division of Medical Imaging and Technology at Karolinska Institutet, Stockholm, Sweden; 2 Department of Medicine, Section of Cardiology, Karolinska University Hospital Huddinge and Karolinska Institutet, Stockholm, Sweden; 3 Cardiology Unit, Department of Medicine, Karolinska University Hospital Solna and Karolinska Institutet, Stockholm, Sweden; 4 Institution for Clinical Science and Education at Södersjukhuset, Karolinska Institutet, Stockholm, Sweden; University of Groningen, Netherlands

## Abstract

**Objectives:**

Patients presenting with acute myocardial infarction and angiographically normal coronary arteries (MINCA) represent a diagnostic and a therapeutic challenge. Cardiac computed tomography (CT) allows detection of coronary artery disease (CAD) even in the absence of significant stenosis. We aimed to investigate whether patients suffering from MINCA had a greater coronary plaque burden, as determined by cardiac CT, than a matched group of healthy volunteers.

**Methods:**

Consecutive patients, aged 45 to 70, with MINCA were enrolled in the Stockholm metropolitan area. Patients with myocarditis were excluded using cardiovascular magnetic resonance imaging. Remaining patients underwent cardiac CT, as did a reference group of healthy volunteers matched by age and gender, with no known cardiovascular disease. Plaque burden was evaluated semi-quantitatively on a per patient and a per segment level.

**Results:**

Despite a higher prevalence of smoking and hypertension, patients with MINCA did not have more CAD than healthy volunteers. Among 57 MINCA patients and 58 volunteers no signs of CAD were found in 24 (42%) and 25 (43%) respectively. On a *per segment* level, MINCA patients had less segments with stenosis ≥20% (2% vs. 5%, p<0.01), as well as a smaller proportion of large (2% vs. 4%, p<0.05) and mixed type plaques (1% vs. 4%, p<0.01). The median coronary calcium score did not differ between MINCA patients and healthy volunteers (6 vs. 8, *ns*).

**Conclusions:**

MINCA patients with no or minimal angiographic stenosis do not have more coronary atherosclerosis than healthy volunteers, and a large proportion of these patients do not have any signs of CAD, as determined by cardiac CT. The MINCA patient group is probably heterogeneous, with a variety of different underlying mechanisms. Non-obstructive CAD is most likely not the most prevalent cause of myocardial infarction in this patient group.

## Introduction

In a considerable number of patients presenting with acute myocardial infarction who undergo conventional coronary angiography, no significant coronary artery stenoses are found. This condition is called MINCA (myocardial infarction and angiographically normal coronary arteries) or MINOCA (myocardial infarction and non-obstructed coronary arteries). Depending on the definition, the reported prevalence of MINCA ranges between 3% and 18% of all acute myocardial infarctions. [1]–[5] In women, however, the prevalence has been reported to be as high as 33%. [4], [5] A number of underlying mechanisms have been proposed, including vasospasm, embolism, disturbed endothelial function and coronary artery disease (CAD) with occult rupture of non-stenotic plaque. [6]–[8] In a clinical setting, Takotsubo cardiomyopathy can be considered a subtype of MINCA. For this condition, often provoked by stress, additional underlying mechanisms have been suggested, such as exaggerated sympathetic stimulation and catecholamine toxicity. [9], [10] Although awareness of MINCA has increased, there is still a lack of data clarifying its different aetiologies and mechanisms.

The development of diagnostic imaging methods has provided new means for understanding MINCA and its underlying causes. The use of cardiovascular magnetic resonance imaging (CMR) for instance, enables differentiation between myocarditis and myocardial infarction, as well as diagnosis of Takotsubo cardiomyopathy. [1], [11] Cardiac computed tomography (CT) has evolved substantially during the last few years, and has now achieved very good accuracy when it comes to diagnosing stenoses of the coronary arteries, as compared to coronary angiography. [12] Cardiac CT has also proven highly sensitive in detecting atherosclerotic plaques in the proximal segments of the coronary arteries, when compared to intravascular ultrasound. [13] An important difference between conventional coronary angiography and cardiac CT is the fact that the former only shows the lumen of the artery, whilst the latter permits visualization of the vascular wall as well as the lumen. Cardiac CT allows detection and characterization of coronary atherosclerotic plaques even when they do not give rise to stenoses and whether they are calcified or not. Accordingly, cardiac CT makes it possible to assess atherosclerotic plaques undetected by conventional coronary angiography.

Previous studies, using intravascular ultrasound or cardiac CT, have suggested that CAD is an important underlying cause of MINCA. [7], [14]–[16] However, none of these studies included a control group. Since other cardiac CT studies including asymtomatic subjects have demonstrated a non negligible prevalence of CAD, the question remains whether the CAD shown in MINCA patients was necessarily the cause of the myocardial infarction. [17]–[19].

The present study is a substudy of the SMINC (Stockholm Myocardial Infarction with Normal Coronaries) study and aims to examine whether patients suffering from MINCA have more coronary atherosclerosis, as assessed by cardiac CT, than a reference group of healthy volunteers [22].

## Materials and Methods

### Ethics Statement

The study conforms to the principles of the Declaration of Helsinki and was approved by the Regional Ethical Review Board in Stockholm (www.epn.se) and by the Radiation Protection Committee of the Karolinska University Hospital. Written informed consent was obtained from all patients and healthy volunteers.

### Study Group

Between June 2007 and May 2011, patients with MINCA were screened for the SMINC study at five different coronary care units in the Stockholm metropolitan area, as described by Collste *et al*. [22] Patients were eligible to take part in the study if they were between 35 and 70 years of age, fulfilled the criteria for acute myocardial infarction according to the universal definition of myocardial infarction, [20] and underwent a coronary angiography showing no or minimal signs of atherosclerosis (defined as the presence of plaque discernible on coronary angiography, but no stenosis exceeding 30% by visual estimation). Coronary angiography was performed at the time of initial hospital admission, according to clinical routines, and evaluated using the modified American Heart Association 17 segment classification. [21] Exclusion criteria were a patient history of structural or coronary heart disease, chronic obstructive lung disease, renal disease, the use of a pacemaker and an electrocardiogram (ECG) on admission showing non sinus rhythm or a clinical diagnosis of pulmonary embolism. CMR was performed on all patients in order to exclude those with myocarditis. [22] After patient inclusion, the coronary angiogram as well as the clinical diagnosis of acute myocardial infarction were re-evaluated by an additional investigator. A reference group of healthy volunteers, matched by age and gender, with no known cardiovascular disease, was recruited using a registry comprising all Stockholm residents. Persons of the same age and gender as MINCA patients were contacted by mail. If they were willing to participate and had no history of cardiovascular disease they underwent an exercise stress test, and if the test was normal they were invited to take part in the study.

Out of 152 patients screened for the SMINC study, 100 MINCA patients were after exclusions described above considered for the present cardiac CT substudy, as well as 100 healthy volunteers. Accordingly, the MINCA patients of the present study form a subgroup of the patients studied by Collste *et al*. [22] Additional exclusion criteria for the CT study were age under 45 (due to considerations of radiation dose; 5 MINCA patients and 5 control persons), previous adverse reaction to iodinated contrast media (1 MINCA patient) and an irregular heart rate (jeopardizing the diagnostic quality of the CT scan; 1 control person). Out of 100 MINCA patients and 100 healthy volunteers, 61 and 58 respectively agreed to take part in the present study. The CT examinations of the MINCA patients were performed between 3 and 6 months after the acute event.

### Cardiac CT Data Acquisition

Examinations were performed on a 64-slice CT scanner (LightSpeed VCT XT; GE Healthcare, Milwaukee, WI, USA). A prospectively ECG-triggered scan protocol was used: detector configuration 64×0.625 mm, rotation time 350 ms, tube potential 120 kV, tube current 450–650 mA (according to patient size). The scans were performed in diastole, in general at 70–75% of the RR interval, with a padding of 100–200 ms, depending on heart rate and variability. The contrast agent used was iodixanol 320 mg I/ml (Visipaque, GE Healthcare, Stockholm, Sweden), which was administered using a dual-head injector (Medrad, Stellant Dual Head Injector, Pittsburgh, PA, USA) and a triple-phase protocol. The contrast agent was individually dosed, based on body weight (400 mg I/kg, 75–100 ml iodixanol), with a fixed injection time (15 s), resulting in an injection rate of 5–7 ml/s. This was followed by a 50 ml mixture of 40% iodixanol and 60% saline and finally by a 50 ml saline chaser. In the absence of contraindications and depending on the initial heart rate, patients received metoprolol (25–100 mg) per os prior to the examination. Patients also received sublingual nitroglycerine (0.4 mg) 4 minutes before the scan.

To assess the coronary calcium score, a non-enhanced scan was performed, using a prospectively ECG-triggered scan protocol: tube potential 120 kV, tube current 200 mA.

### Cardiac CT Data Analysis

The Cardiac CT exam was analysed independently by three readers (two experienced readers with level 2 and one reader with level 1 according to ACCF/AHA levels of competence), [23] who were blinded to all clinical information. A subsequent joint reading was performed and a consensus reached.

Cardiac CT data analysis was performed using the CardIQ Xpress software on the Advantage Workstation 4.4 (GE Healthcare, Milwaukee, WI, USA). Axial source images, multiplanar and curved multiplanar reformats as well as thin-slab maximum intensity projections were used. The optimal image display setting for lumen and plaque assessment was chosen on an individual basis, but in general at a window width of 800–1000 HU and a level of 100–200 HU. Coronary arteries were subdivided into 17 segments, according to the modified American Heart Association classification. [21] Initially, each segment was assessed regarding image quality and evaluability. Segments were considered non-evaluable if artifacts prevented reliable assessment of the lumen or the vessel wall (e. g. due to motion, image noise or heavy calcification). Secondly, each segment was visually evaluated with regard to the presence of stenosis (A), plaque size (B) and composition (C). A plaque was defined as any structure, discernible in at least two planes, within or adjacent to the vessel lumen, which could be clearly separated from the vessel lumen and from adjacent soft tissue.

- Lesions were quantified for stenosis by visual estimation, comparing the minimal lumen of the stenotic area with the lumen of the adjacent proximal unaffected segment, and expressed in terms of diameter stenosis: <20%, 20–50% or ≥50%.
- The size of the atherosclerotic plaque was determined by measuring the length of the plaque on longitudinal sections and arbitrarily classified as: small (<4 mm), medium (4–8 mm) or large (≥8 mm).
- Plaque composition was visually assessed based on the presence or absence of calcified elements: non-calcified coronary artery plaque, mixed coronary artery plaque or calcified coronary artery plaque, the latter with ≥50% calcified tissue. [24].

In the case of more than one atherosclerotic plaque in a single segment, only the greatest degree of stenosis, the largest plaque size and the most pronounced calcification was considered.

### Coronary Calcium Score

The coronary calcium score was calculated using semi-automatic software (SmartScore 4.0, GE Healthcare, Milwaukee, WI, USA) on the Advantage Workstation 4.4 (GE Healthcare, Milwaukee, WI, USA). The total calcium burden of the coronary arteries was reported in terms of AJ-130 score, based on the scoring algorithm of Agatston et al. [25].

### Statistical Analysis

In order to evaluate hypotheses of variables in contingency tables, the chi-square test was used or, in the case of small expected frequencies, Fisher’s exact two-sided test. Statistical comparisons for testing differences between two independent groups were made using the Student’s t-test for uncorrelated means, after validation of normal distribution by use of the Shapiro Wilk test. In the case of non-normal distribution the Mann-Whitney test was used. In addition, descriptive statistics was used to characterize the data. All analyses were carried out using the SAS system and the 5% levels of significance were considered. In the case of a statistically significant result the probability value (p-value) has been given. Due to the nature of the study, an initial exploratory study, the sample size was not determined based on power or clinical difference. The number of patients participating in the study was chosen for practical reasons, not statistical. However, in order to estimate statistical power, one could consider the per segment analysis (n = 765+781) and a binomial endpoint (segment with or without CAD) and a 5% level of significance. Anticipating a prevalence of CAD of 10% in the control group and 15% in the MINCA group would yield a power of 85%.

## Results

**Figure 1:**
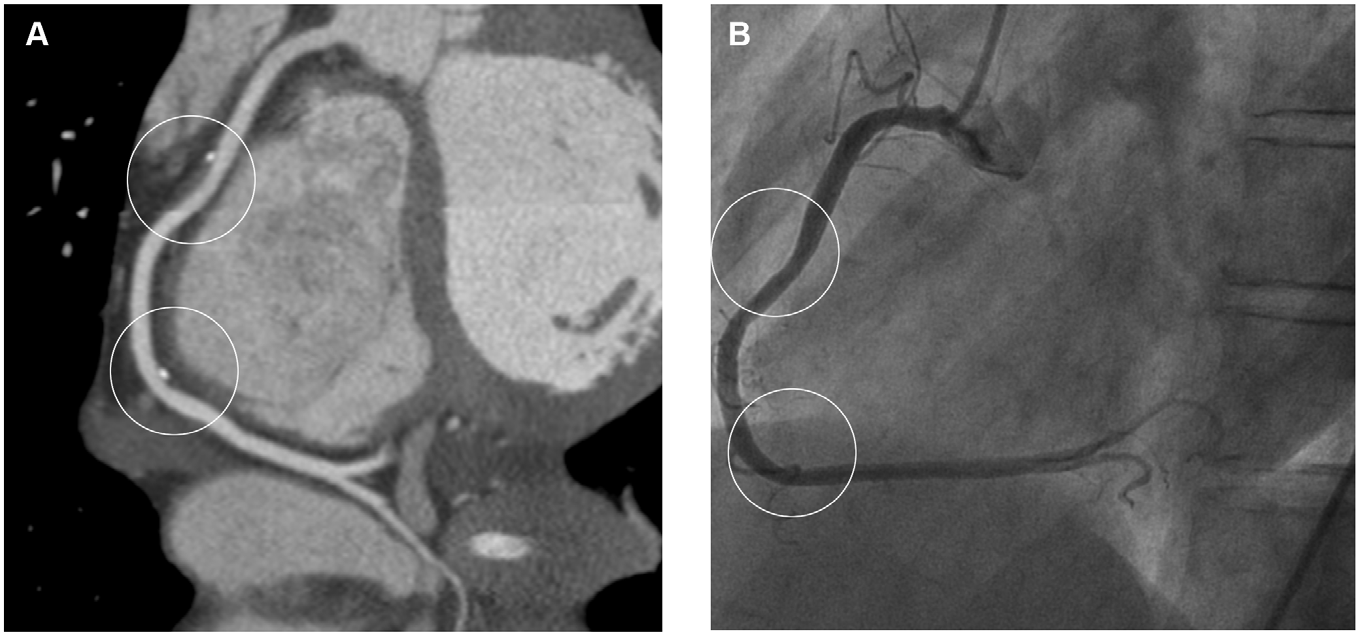
The right coronary artery in a patient presenting with acute myocardial infarction. Cardiac computed tomography (A) shows a large atherosclerotic plaque and more distally a small plaque, both with <20% stenosis. Coronary angiography (B) shows only minimal signs of atherosclerosis.

**Figure 2:**
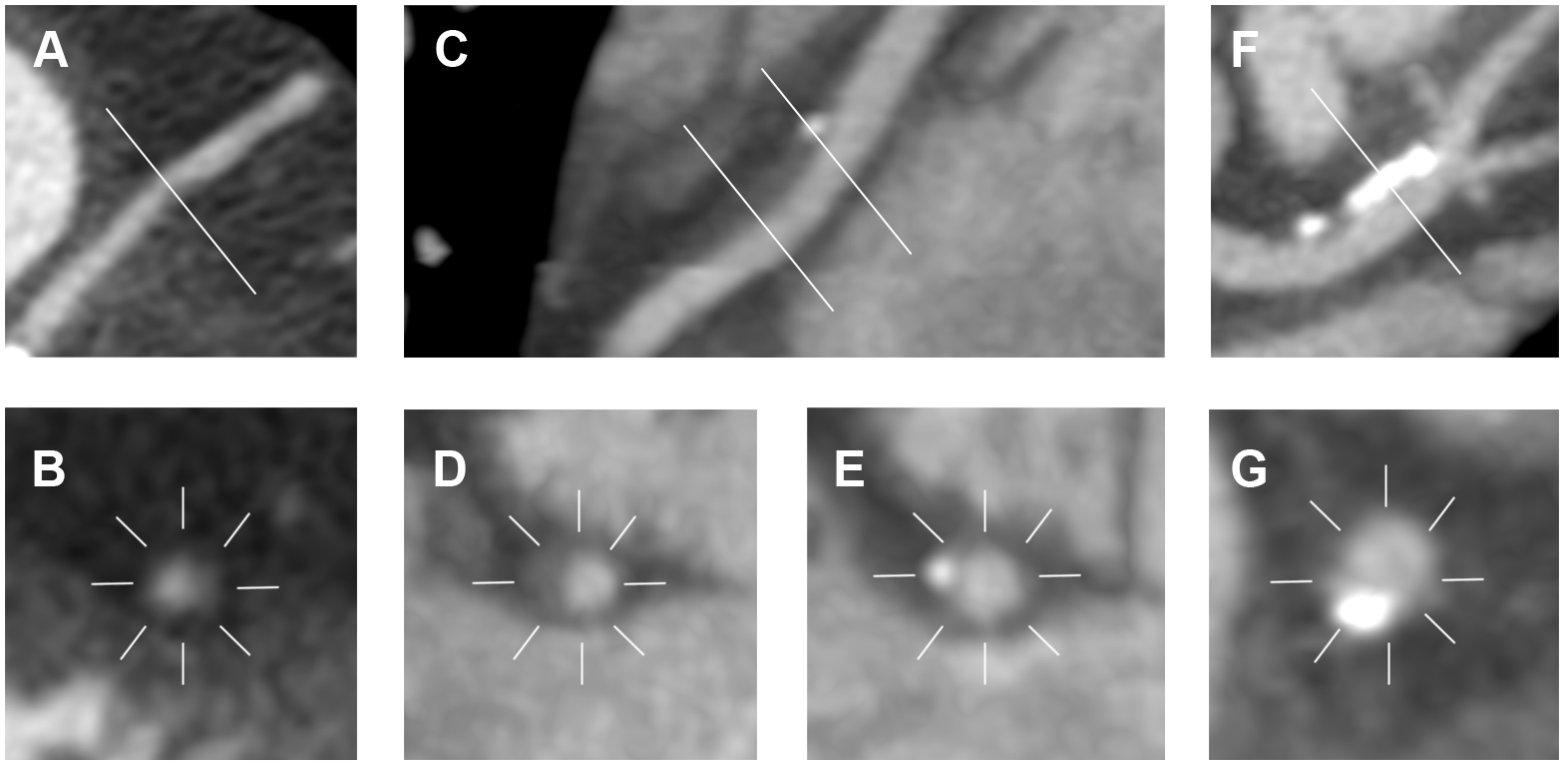
Different plaque types, as seen by cardiac computed tomography. A non-calcified plaque is shown in longitudinal and cross section (A and B). The degree of stenosis was 20–50%. A large mixed plaque is shown in longitudinal section (C) and in cross section at the level of non calcified (D) and calcified (E) components. A large calcified plaque is shown to the right. (F and G). The mixed and calcified plaques (C to G) were both eccentric in location and the degree of stenosis was <20%.

Of the 119 (61 MINCA+58 volunteers) cardiac CT exams performed, 4 exams of MINCA patients had to be excluded, 3 due to motion artifacts resulting in <7 evaluable segments and 1 due to the heart not being completely covered by the scan. Thus, cardiac CT exams of 57 MINCA patients and 58 healthy volunteers were further analyzed. From these exams 15 (1.9%) individual segments in the MINCA group and 11 (1.4%) in the reference group were non-evaluable and excluded from analyses. Figure 1 shows an example of a coronary artery in a MINCA patient, as visualized by cardiac CT and by conventional coronary angiography. Figure 2 shows examples of different plaque types as seen by cardiac CT.

Baseline characteristics of patients with MINCA and healthy volunteers are compared in Table 1. Current smoking and treated hypertension were more common in the MINCA group. Regarding all other variables the two groups were comparable. Out of 57 MINCA patients, 56 presented with no signs of heart failure (Killip class 1) and only one with heart failure, consistent with Killip class 2. Signs of acute ischemia (ST-T changes or left bundle branch block) on admission ECG were present in 31 (54%) MINCA patients, of whom 10 had ST elevations. The median (IQR) maximum troponin level was 18 (7–43) times greater than the upper limit of normal. Myocardial infarction was detectable with CMR in 11 (19%) patients. The criteria for Takotsubo cardiomyopathy was fulfilled in 15 (26%).

**Table 1:** Baseline characteristics.

	MINCA, n = 57	Healthy subjects, n = 58
Age (years)	60±5	61±6
Female	42 (74%)	39 (67%)
Present smoking	10 (18%)	2 (3%)*
Prior smoking	17 (30%)	23 (40%)
Family history of CAD	16 (28%)	14 (24%)
Diabetes mellitus	1 (2%)	0 (0%)
Treated hypertension	19 (33%)	6 (10%)†
Treated hyperlipidemia	8 (14%)	3 (5%)
BMI (kg/m^2^)	25.8±3	25.8±3

Abbreviations: MINCA, myocardial infarction with angiographically normal coronary arteries; CAD, coronary artery disease; BMI, body mass index; SD, standard deviation. Data are presented as mean ± SD or absolute value (percentage).

*P<0.05,

† P<0.01, using Fisher’s exact test.

The cardiac CT plaque burden analyses are presented in Tables 2 and 3, on per patient (Table 2) and per segment (Table 3) basis, comparing the MINCA group with the reference group. On a *per patient* level there were no statistically significant differences in severity or extent of CAD. Twentyfour (42%) MINCA patients and 25 (43%) healthy subjects had no signs of CAD. When analyzing the data on a *per segment* level, however, there were statistically significant differences regarding degree of stenosis, plaque size and plaque composition. MINCA patients had less segments with stenosis ≥20% compared to healthy volunteers (2% vs 5%, p<0.01) They also exhibited a smaller proportion of large plaques (2% vs 4%, p<0.05) and mixed type coronary artery plaques (1% vs 4%, p<0.01). The calcium scores within each group were diverse, but no significant differences were found between the groups. No differences were found regarding CT plaque burden when MINCA patients with and without MI detected by CMR were compared or when MINCA patients with and without ST elevations were compared (Table S1). Nor were there any differences in plaque burden between MINCA patients with and without a diagnosis of Takotsubo cardiomyopathy (Table S1). No statistically significant difference was demonstrated regarding peak troponin levels between MINCA patients with and without CAD. There were no differences in terms of baseline characteristics when MINCA patients without CAD were compared to those with CAD demonstrated by cardiac CT (Table S2).

**Table 2:** Cardiac CT plaque burden per patient.

		MINCA patients,n = 57	Healthy volunteers,n = 58	*P*
Severity of CAD*	No CAD	24 (42%)	25 (43%)	*ns*
	Stenosis <20%	22 (39%)	23 (40%)	
	Stenosis 20–50%	11 (19%)	9 (16%)	
	Stenosis ≥50%	0 (0%)	1 (2%)	
All CAD†	0 segments	24 (42%)	25 (43%)	*ns*
	1 segments	14 (25%)	10 (17%)	
	2 segments	8 (14%)	12 (21%)	
	3 segments	4 (7%)	6 (10%)	
	4 segments	2 (4%)	0 (0%)	
	5 segments	3 (5%)	1 (2%)	
	6 segments	0 (0%)	0 (0%)	
	7 segments	0 (0%)	0 (0%)	
	8 segments	2 (4%)	1 (2%)	
	9 segments	0 (0%)	1 (2%)	
	10 segments	0 (0%)	2 (3%)	
Calcium score (AJ-130)		6 (0–778)	8 (0–1882)	*ns*

Abbreviations: Cardiac CT, cardiac computed tomography; MINCA, myocardial infarction with angiographically normal coronary arteries; CAD, coronary artery disease; *ns*, non significant. Values are presented as absolute value (percentage) or median (range).

*refers to the maximum diameter stenosis;

† refers to obstructive and non-obstructive CAD.

**Table 3:** Cardiac CT plaque burden per segment.

		MINCA patients,765 segments	Healthy volunteers,781 segments	*P*
Severity of CAD	No CAD	684 (89%)	687 (88%)	<0.01*
	Stenosis <20%	68 (9%)	58 (7%)	
	Stenosis 20–50%	13 (2%)	35 (4%)	
	Stenosis ≥50%	0 (0%)	1 (0.1%)	
Plaque size	No CAD	684 (89%)	687 (88%)	0.04*
	Small	41 (5%)	31 (4%)	
	Medium	24 (3%)	29 (4%)	
	Large	16 (2%)	34 (4%)	
Plaque composition	No CAD	684 (89%)	687 (88%)	0.04*
	Non-calcified plaque	10 (1%)	10 (1%)	
	Mixed plaque	10 (1%)	28 (4%)	
	Calcified plaque	61 (8%)	56 (7%)	

Abbreviations: Cardiac CT, cardiac computed tomography; MINCA, myocardial infarction with angiographically normal coronary arteries; CAD, coronary artery disease; Values are presented as absolute value (percentage).

*P-values apply to the comparison of the four categories in the two columns to the left of the value, using the chi-square test.

## Discussion

This study is the first to analyze coronary plaque burden in patients with MINCA by means of cardiac CT, with a matched reference group for comparison. The most important finding was the fact that patients with MINCA did not have more coronary atherosclerosis than healthy volunteers, despite a higher frequency of smoking and hypertension in the MINCA group. In addition, a high proportion of MINCA patients (42%) did not exhibit any signs of CAD, as demonstrated by cardiac CT, which strongly suggests that there are underlying causes other than CAD in a significant number of MINCA patients. A finding that further strengthens this hypothesis was the fact that MINCA patients had a lower rate of large size atherosclerotic plaques and mixed type coronary artery plaques compared to healthy volunteers. Such plaque characteristics have been shown to imply a more vulnerable plaque, more prone to rupture [26]–[28].

In the literature there are several definitions and terms used to designate myocardial infarction in the absence of significant CAD. The term MINOCA (myocardial infarction and non-obstructed coronary arteries) is often used, since many studies have included patients with <50% angiographic stenosis. However, the term MINCA has been chosen for this study, in order to stress the fact that only patients with no or minimal signs of atherosclerosis on coronary angiography were included.

The findings of the current study partly contradict findings of a recent study by Aldrovandi *et al.*, where only 16% of MINCA patients had no signs of CAD when examined with cardiac CT. [16] However, this difference can probably be explained by different inclusion criteria. In the previous study patients with <50% angiographic diameter stenosis were included, whereas in the current study a more rigorous definition was used, including only patients with no or minimal signs of atherosclerosis. The mean degree of stenosis, as determined by cardiac CT, was >30% in the previous study, whereas the median degree of stenosis in the present study was <20%. Hence, applying a more rigorous definition of “normal coronary angiogram” seems useful in order to identify a patient group that is far less likely to have CAD. In addition, only patients with evidence of myocardial infarction on CMR were included in the study by Aldrovandi *et al*., whereas the present study also included patients with smaller myocardial infarctions, proven by biochemical markers but not detectable by CMR.

A study by Reynolds *et al.* supports that CAD is a major cause of myocardial infarction in patients with higher degrees of non-obstructive stenosis (<50%) at coronary angiography, but not necessarily in patients with no or minimal signs of atherosclerosis. [7] In their study women with myocardial infarction without angiographically obstructive CAD underwent intravascular ultrasound, which revealed plaque ruptures and ulcerations in 38% of patients. A higher degree of stenosis was found in patients with plaque disruption (median degree of stenosis 40%) compared to patients without plaque disruption. One third of patients had no signs of atherosclerosis on coronary angiography and, interestingly, none of these patients had plaque disruption. Consequently, it seems likely that the frequency of plaque disruption would be smaller in the present study group, with less severe CAD.

This study cannot explain the underlying cause of MINCA. One explanation could be that an important proportion of MINCA cases are in fact Takotsubo cardiomyopathy, which was found in 26% of the study group. For the MINCA patients who had CAD, this may at least for some patients have been the cause of the myocardial infarction. Plaque disruption with transient thrombus formation might be one plausible mechanism and vasospasm another. [7] There was a higher frequency of smoking in the MINCA group, which may in turn increase the risk of thrombosis, as well as vasoconstriction. An increased resistance to activated protein C has been reported in MINCA patients, which supports the theory that thrombosis is involved in the aetiology of MINCA [4].

The present study has limitations. Although being the largest study of its kind, the sample size was limited. Thus, lack of significant differences may still be caused by lack of power to detect such differences. However, the data show a tendency towards more severe CAD in the reference group compared to the MINCA-group, rather than the other way round.

The fact that MINCA patients, on a per segment level, showed a lower frequency of stenoses ≥20% than healthy subjects can probably be explained by the selection process, where MINCA patients per definition had no or minimal stenosis as determined by conventional coronary angiography. One could argue that the reference group should have been selected by excluding individuals with angiographic coronary stenoses. This was however not achievable, since it is not ethically possible to perform invasive coronary angiography on healthy volunteers. Still, on a per patient level there were roughly equal numbers of MINCA patients (19%) and healthy controls (18%) with stenosis ≥20%. Only 57 of the initial 100 MINCA patients were included in the cardiac CT analyses. There were however no differences in baseline characteristics when these patients were compared with the 43 MINCA patients who did not participate in the cardiac CT study (Table S3). The study population mainly reflects a northern European ethnic group, the majority being women, which might affect generalizability. However, the female predominance is not surprising, since it reflects the higher prevalence of MINCA in women compared to men. Although 64 detector cardiac CT has proven highly sensitive in detecting atherosclerotic plaques, its spatial resolution is limited, which makes it hazardous to assess very small structures. A study comparing cardiac CT plaque detection and quantification with IVUS measures showed a very good diagnostic accuracy for cardiac CT to detect atherosclerotic plaques on a segmental level. However, the sensitivity decreased for smaller plaques and for plaques located more distally in the coronary arteries. [29] Hence, early coronary atherosclerosis, with very subtle changes of the vessel wall, as well as distal lesions might remain undetected by cardiac CT. For the MINCA patients there was a time span of 3 to 6 months between the acute event and the CT examination, which might have had minor influence on the results. A semi-quantitative method was used to estimate plaque burden. It could be debated whether an automated, non-user dependent method should have been used. However, such automated approaches have not been thoroughly validated, whilst semi-quantitative methods relying on visual assessment for plaque detection and characterization have been described in several studies and have been found to entail good intra- and interobserver variability [30].

The current findings together with findings of previous studies suggest that patients with MINCA compose a heterogeneous group, with a variety of underlying causes of the myocardial infarction. It seems that in patients with moderate angiographic coronary stenosis, CAD with plaque disruption might be an important cause of the myocardial infarction, whilst in patients with no or minimal angiographic stenosis the myocardial infarction is more likely to have other causes than established CAD. It is now well recognized that atherosclerosis is a heterogeneous process, including for instance inflammation and endothelial dysfunction, and additional research is warranted in order to investigate the role of these processes in the MINCA patient group.

Since the prognosis for patients suffering from MINCA is not benign, [31], [32] it is of great importance to further clarify the underlying mechanisms, in order to offer patients appropriate treatment and care.

## Supporting Information

Table S1 Cardiac CT plaque burden, comparing subgroups of MINCA patients.
(PDF)

Table S2 Baseline characteristics for MINCA patients with and without CAD.
(PDF)

Table S3 Baseline characteristics for MINCA patients that participated and did not participate in the CT substudy.
(PDF)

## References

[1] Stensaeth KH, Fossum E, Hoffmann P, Mangschau A, Klow NE (2011) Clinical characteristics and role of early cardiac magnetic resonance imaging in patients with suspected ST-elevation myocardial infarction and normal coronary arteries. Int J Cardiovasc Imaging 27: 355–365. 20652637 10.1007/s10554-010-9671-7 PMC3092060

[2] Gehrie ER, Reynolds HR, Chen AY, Neelon BH, Roe MT, et al (2009) Characterization and outcomes of women and men with non-ST-segment elevation myocardial infarction and nonobstructive coronary artery disease: Results from the can rapid risk stratification of unstable angina patients suppress adverse outcomes with early implementation of the ACC/AHA guidelines (CRUSADE) quality improvement initiative. Am Heart J 158: 688–694. 19781432 10.1016/j.ahj.2009.08.004

[3] Larsen AI, Galbraith PD, Ghali WA, Norris CM, Graham MM, et al (2005) Characteristics and outcomes of patients with acute myocardial infarction and angiographically normal coronary arteries. Am J Cardiol 95: 261–263. 15642564 10.1016/j.amjcard.2004.09.014

[4] Agewall S, Daniel M, Eurenius L, Ekenback C, Skeppholm M, et al (2012) Risk factors for myocardial infarction with normal coronary arteries and myocarditis compared with myocardial infarction with coronary artery stenosis. Angiology 63: 500–503. 22210737 10.1177/0003319711429560

[5] Chokshi NP, Iqbal SN, Berger RL, Hochman JS, Feit F, et al (2010) Sex and race are associated with the absence of epicardial coronary artery obstructive disease at angiography in patients with acute coronary syndromes. Clin Cardiol 33: 495–501. 20734447 10.1002/clc.20794 PMC6653646

[6] Ong P, Athanasiadis A, Hill S, Vogelsberg H, Voehringer M, et al (2008) Coronary artery spasm as a frequent cause of acute coronary syndrome: The CASPAR (coronary artery spasm in patients with acute coronary syndrome) study. J Am Coll Cardiol 52: 523–527. 18687244 10.1016/j.jacc.2008.04.050

[7] Reynolds HR, Srichai MB, Iqbal SN, Slater JN, Mancini GBJ, et al (2011) Mechanisms of myocardial infarction in women without angiographically obstructive coronary artery disease. Circulation 124: 1414–1425. 21900087 10.1161/CIRCULATIONAHA.111.026542 PMC3619391

[8] Kardasz I, De Caterina R (2007) Myocardial infarction with normal coronary arteries: A conundrum with multiple aetiologies and variable prognosis: An update. J Intern Med 261: 330–348. 17391108 10.1111/j.1365-2796.2007.01788.x

[9] Wittstein IS, Thiemann DR, Lima JA, Baughman KL, Schulman SP, et al (2005) Neurohumoral features of myocardial stunning due to sudden emotional stress. N Engl J Med 352: 539–548. 15703419 10.1056/NEJMoa043046

[10] Prasad A, Lerman A, Rihal CS (2008) Apical ballooning syndrome (tako-tsubo or stress cardiomyopathy): A mimic of acute myocardial infarction. Am Heart J 155: 408–417. 18294473 10.1016/j.ahj.2007.11.008

[11] Laissy JP, Hyafil F, Feldman LJ, Juliard JM, Schouman-Claeys E, et al (2005) Differentiating acute myocardial infarction from myocarditis: Diagnostic value of early- and delayed-perfusion cardiac MR imaging. Radiology 237: 75–82. 16126925 10.1148/radiol.2371041322

[12] Mowatt G, Cook JA, Hillis GS, Walker S, Fraser C, et al (2008) 64-slice computed tomography angiography in the diagnosis and assessment of coronary artery disease: Systematic review and meta-analysis. Heart 94: 1386–1393. 18669550 10.1136/hrt.2008.145292

[13] Voros S, Rinehart S, Qian Z, Joshi P, Vazquez G, et al (2011) Coronary atherosclerosis imaging by coronary CT angiography: Current status, correlation with intravascular interrogation and meta-analysis. JACC Cardiovasc Imaging 4: 537–548. 21565743 10.1016/j.jcmg.2011.03.006

[14] Caussin C, Ohanessian A, Lancelin B, Rahal S, Hennequin R, et al (2003) Coronary plaque burden detected by multislice computed tomography after acute myocardial infarction with near-normal coronary arteries by angiography. Am J Cardiol 92: 849–852. 14516892 10.1016/s0002-9149(03)00899-3

[15] Aldrovandi A, Cademartiri F, Menozzi A, Ugo F, Lina D, et al (2008) Evaluation of coronary atherosclerosis by multislice computed tomography in patients with acute myocardial infarction and without significant coronary artery stenosis a comparative study with quantitative coronary angiography. Circ Cardiovasc Imaging 1: 205–211. 19808544 10.1161/CIRCIMAGING.108.786962

[16] Aldrovandi A, Cademartiri F, Arduini D, Lina D, Ugo F, et al (2012) Computed tomography coronary angiography in patients with acute myocardial infarction without significant coronary stenosis. Circulation 126: 3000–3007. 23168414 10.1161/CIRCULATIONAHA.112.117598

[17] Hwang Y, Kim Y, Chung IM, Ryu J, Park H (2010) Coronary heart disease risk assessment and characterization of coronary artery disease using coronary CT angiography: Comparison of asymptomatic and symptomatic groups. Clin Radiol 65: 601–608. 20599061 10.1016/j.crad.2010.04.009

[18] Hadamitzky M, Meyer T, Hein F, Bischoff B, Martinoff S, et al (2010) Prognostic value of coronary computed tomographic angiography in asymptomatic patients. Am J Cardiol 105: 1746–1751. 20538125 10.1016/j.amjcard.2010.01.354

[19] Neefjes LA, Ten Kate GJ, Rossi A, Galema-Boers AJ, Langendonk JG, et al (2011) CT coronary plaque burden in asymptomatic patients with familial hypercholesterolaemia. Heart 97: 1151–1157. 21565855 10.1136/hrt.2010.220699

[20] Thygesen K, Alpert JS, White HD, Jaffe AS, Apple FS, et al (2007) Universal definition of myocardial infarction. Circulation 116: 2634–2653. 17951284 10.1161/CIRCULATIONAHA.107.187397

[21] Austen WG, Edwards JE, Frye RL, Gensini GG, Gott VL, et al (1975) A reporting system on patients evaluated for coronary artery disease. Report of the ad hoc committee for grading of coronary artery disease, council on cardiovascular surgery, american heart association. Circulation 51: 5–40. 1116248 10.1161/01.cir.51.4.5

[22] Collste O, Sorensson P, Frick M, Agewall S, Daniel M, et al (2013) Myocardial infarction with normal coronary arteries is common and associated with normal findings on cardiovascular magnetic resonance imaging: Results from the stockholm myocardial infarction with normal coronaries study. J Intern Med 273: 189–196. 22742529 10.1111/j.1365-2796.2012.02567.x

[23] Budoff MJ, Cohen MC, Garcia MJ, Hodgson JM, Hundley WG, et al (2005) ACCF/AHA clinical competence statement on cardiac imaging with computed tomography and magnetic resonance. Circulation 112: 598–617. 16046290 10.1161/CIRCULATIONAHA.105.168237

[24] Leber AW, Knez A, Becker A, Becker C, von Ziegler F, et al (2004) Accuracy of multidetector spiral computed tomography in identifying and differentiating the composition of coronary atherosclerotic plaques: A comparative study with intracoronary ultrasound. J Am Coll Cardiol 43: 1241–1247. 15063437 10.1016/j.jacc.2003.10.059

[25] Agatston AS, Janowitz WR, Hildner FJ, Zusmer NR, Viamonte M Jr, et al (1990) Quantification of coronary artery calcium using ultrafast computed tomography. J Am Coll Cardiol 15: 827–832. 2407762 10.1016/0735-1097(90)90282-t

[26] Pundziute G, Schuijf JD, Jukema JW, Decramer I, Sarno G, et al (2008) Evaluation of plaque characteristics in acute coronary syndromes: Non-invasive assessment with multi-slice computed tomography and invasive evaluation with intravascular ultrasound radiofrequency data analysis. Eur Heart J 29: 2373–2381. 18682447 10.1093/eurheartj/ehn356

[27] Pflederer T, Marwan M, Schepis T, Ropers D, Seltmann M, et al (2010) Characterization of culprit lesions in acute coronary syndromes using coronary dual-source CT angiography. Atherosclerosis 211: 437–444. 20189568 10.1016/j.atherosclerosis.2010.02.001

[28] Virmani R, Burke AP, Farb A, Kolodgie FD (2006) Pathology of the vulnerable plaque. J Am Coll Cardiol 47: C13–18. 16631505 10.1016/j.jacc.2005.10.065

[29] Papadopoulou SL, Neefjes LA, Schaap M, Li HL, Capuano E, et al (2011) Detection and quantification of coronary atherosclerotic plaque by 64-slice multidetector CT: A systematic head-to-head comparison with intravascular ultrasound. Atherosclerosis 219: 163–170. 21802687 10.1016/j.atherosclerosis.2011.07.005

[30] Maurovich-Horvat P, Ferencik M, Bamberg F, Hoffmann U (2009) Methods of plaque quantification and characterization by cardiac computed tomography. J Cardiovasc Comput Tomogr 3 Suppl 2 S91–98. 20129522 10.1016/j.jcct.2009.10.012

[31] Bugiardini R, Manfrini O, De Ferrari GM (2006) Unanswered questions for management of acute coronary syndrome: Risk stratification of patients with minimal disease or normal findings on coronary angiography. Arch Intern Med 166: 1391–1395. 16832004 10.1001/archinte.166.13.1391

[32] Larsen AI, Nilsen DW, Yu J, Mehran R, Nikolsky E, et al (2013) Long-term prognosis of patients presenting with ST-segment elevation myocardial infarction with no significant coronary artery disease (from the HORIZONS-AMI trial). Am J Cardiol 111: 643–648. 23261001 10.1016/j.amjcard.2012.11.011

