# Supplementary material for: Coronary Plaque Burden, as Determined by Cardiac Computed Tomography, in Patients with Myocardial Infarction and Angiographically Normal Coronary Arteries Compared to Healthy Volunteers: A Prospective Multicenter Observational Study
Source: PLoS One. 2014 Jun 17;9(6):e99783. doi: 10.1371/journal.pone.0099783 (PMC4061030; doi:10.1371/journal.pone.0099783)
Supplement: Table S1 — Cardiac CT plaque burden, comparing subgroups of MINCA patients. (PDF) [file pone.0099783.s001.pdf]

Table S1. Cardiac CT plaque burden per patient and per segment, comparing subgroups of MINCA patients

	MINCA (all)	MINCA (Takotsubo)	MINCA (Takotsubo excluded)	MINCA (MI detected by CMR)	MINCA (no MI detected by CMR)	MINCA (ST-elevation)	MINCA (without ST-elevation)	Healthy volunteers
Patients	n=57	n=15	n= 42	n=11	n=46	n=10	n=47	n=58
No CAD	24 (42%)	7 (47%)	17 (40%)	5 (45%)	19 (41%)	4 (40%)	20 (43%)	25 (43%)
Stenosis <20%	22 (39%)	5 (33%)	17 (40%)	5 (45%)	17 (37%)	3 (30%)	19 (40%)	23 (40%)
Stenosis 20-50%	11 (19%)	3 (20%)	8 (19%)	1 (9%)	10 (22%)	3 (30%)	8 (17%)	9 (16%)
Stenosis ≥50%	0 (0%)	0 (0%)	0 (0%)	0 (0%)	0 (0%)	0 (0%)	0 (0%)	1 (2%)
0 segments	24 (42%)	7 (47%)	17 (40%)	5 (45%)	19 (41%)	4 (40%)	20 (43%)	25 (43%)
1 segments	14 (25%)	6 (40%)	8 (19%)	2 (18%)	12 (26%)	3 (30%)	11 (23%)	10 (17%)
2 segments	8 (14%)	0 (0%)	8 (19%)	3 (27%)	5 (11%)	0 (0%)	8 (17%)	12 (21%)
3 segments	4 (7%)	0 (0%)	4 (10%)	0 (0%)	4 (9%)	2 (20%)	2 (4%)	6 (10%)
4 segments	2 (4%)	0 (0%)	2 (5%)	0 (0%)	2 (4%)	0 (0%)	2 (4%)	0 (0%)
5 segments	3 (5%)	1 (7%)	2 (5%)	0 (0%)	3 (7%)	1 (10%)	2 (4%)	1 (2%)
6 segments	0 (0%)	0 (0%)	0 (0%)	0 (0%)	0 (0%)	0 (0%)	0 (0%)	0 (0%)
7 segments	0 (0%)	0 (0%)	0 (0%)	0 (0%)	0 (0%)	0 (0%)	0 (0%)	0 (0%)
8 segments	2 (4%)	1 (7%)	1 (2%)	1 (9%)	1 (2%)	0 (0%)	2 (4%)	1 (2%)
9 segments	0 (0%)	0 (0%)	0 (0%)	0 (0%)	0 (0%)	0 (0%)	0 (0%)	1 (2%)
10 segments	0 (0%)	0 (0%)	0 (0%)	0 (0%)	0 (0%)	0 (0%)	0 (0%)	2 (3%)
Segments	n=765	n=207	n=558	n=150	n=615	n=143	n=622	n=781
No CAD	684 (89%)	188 (91%)	496 (89%)	134 (89%)	550 (89%)	129 (90%)	555 (89%)	687 (88%)
Stenosis <20%	68 (9%)	16 (8%)	52 (9%)	15 (10%)	53 (9%)	10 (7%)	58 (9%)	58 (7%)
Stenosis 20-50%	13 (2%)	3 (1%)	10 (2%)	1 (1%)	12 (2%)	4 (3%)	9 (1%)	35 (4%)
Stenosis ≥50%	0 (0%)	0 (0%)	0 (0%)	0 (0%)	0 (0%)	0 (0%)	0 (0%)	1 (0.1%)

Abbreviations: Cardiac CT, cardiac computed tomography; MINCA, myocardial infarction and angiographically normal coronary arteries; MI, myocardial infarction; CMR, cardiovascular magnetic resonance imaging; CAD, coronary artery disease. Values are presented as absolute value (percentage).
