# Supplementary material for: Coronary Plaque Burden, as Determined by Cardiac Computed Tomography, in Patients with Myocardial Infarction and Angiographically Normal Coronary Arteries Compared to Healthy Volunteers: A Prospective Multicenter Observational Study
Source: PLoS One. 2014 Jun 17;9(6):e99783. doi: 10.1371/journal.pone.0099783 (PMC4061030; doi:10.1371/journal.pone.0099783)
Supplement: Table S2 — Baseline characteristics for MINCA patients with and without CAD. (PDF) [file pone.0099783.s002.pdf]

Table S2. Baseline characteristics for MINCA patients with and without CAD detected by Cardiac CT

	MINCA with CAD n=33	MINCA without CAD n=24
Age (years)	60 ± 6	59 ± 5
Female	22 (67%)	20 (83%)
Present smoking	5 (15%)	5 (21%)
Prior smoking	12 (36%)	5 (21%)
Family history of CAD	10 (30%)	6 (25%)
Diabetes mellitus	1 (3%)	0 (0%)
Treated hypertension	13 (39%)	6 (25%)
Treated hyperlipidemia	6 (18%)	2 (8%)
BMI (kg/m^2)	26.8 ± 3	24.5 ± 3*

Abbreviations: MINCA, myocardial infarction with angiographically normal coronary arteries; CAD, coronary artery disease; Cardiac CT, cardiac computed tomography; BMI, body mass index; SD, standard deviation. Data are presented as mean ± SD or absolute value (percentage). *P <0.05 using Student´s t-test. No other statistically significant differences in baseline characteristics were found.
