# Supplementary material for: Coronary Plaque Burden, as Determined by Cardiac Computed Tomography, in Patients with Myocardial Infarction and Angiographically Normal Coronary Arteries Compared to Healthy Volunteers: A Prospective Multicenter Observational Study
Source: PLoS One. 2014 Jun 17;9(6):e99783. doi: 10.1371/journal.pone.0099783 (PMC4061030; doi:10.1371/journal.pone.0099783)
Supplement: Table S3 — Baseline characteristics for MINCA patients that participated and did not participate in the CT substudy. (PDF) [file pone.0099783.s003.pdf]

Table S3. Baseline characteristics for MINCA patients that participated and did not participate in CT substudy

	MINCA included n=57	MINCA not included n=43
Age (years)	60 ± 5	58 ± 9
Female	42 (74%)	30 (70%)
Present smoking	10 (18%)	10 (23%)
Prior smoking	17 (30%)	12 (28%)
Diabetes mellitus	1 (2%)	3 (7%)
Treated hypertension	19 (33%)	19 (44%)
Treated hyperlipidemia	8 (14%)	2 (5%)
BMI (kg/m^2)	25.8 ± 3	25.0 ± 4

Abbreviations: MINCA, myocardial infarction with angiographically normal coronary arteries; CT, computed tomography; BMI, body mass index; SD, standard deviation. Data are presented as mean ± SD or absolute value (percentage). No statistically significant differences in baseline characteristics were found.
